# Magnetic Resonance Imaging Is Sensitive to Pathological Amelioration in a Model for Laminin-Deficient Congenital Muscular Dystrophy (MDC1A)

**DOI:** 10.1371/journal.pone.0138254

**Published:** 2015-09-17

**Authors:** Ravneet Vohra, Anthony Accorsi, Ajay Kumar, Glenn Walter, Mahasweta Girgenrath

**Affiliations:** 1 Department of Health Sciences, Sargent College, Boston University, Boston, MA, United States of America; 2 Department of Physiology and Functional Genomics, College of Medicine, University of Florida, Gainesville, FL, United States of America; University of Louisville School of Medicine, UNITED STATES

## Abstract

**Purpose:**

To elucidate the reliability of MRI as a non-invasive tool for assessing in vivo muscle health and pathological amelioration in response to Losartan (Angiotensin II Type 1 receptor blocker) in DyW mice (mouse model for Laminin-deficient Congenital Muscular Dystrophy Type 1A).

**Methods:**

Multiparametric MR quantifications along with histological/biochemical analyses were utilized to measure muscle volume and composition in untreated and Losartan-treated 7-week old DyW mice.

**Results:**

MRI shows that DyW mice have significantly less hind limb muscle volume and areas of hyperintensity that are absent in WT muscle. DyW mice also have significantly elevated muscle levels (suggestive of inflammation and edema). Muscle T_2_ returned to WT levels in response to Losartan treatment. When considering only muscle pixels without T_2_ elevation, DyW T_2_ levels are significantly lower than WT (suggestive of fibrosis) whereas Losartan-treated animals do not demonstrate this decrease in muscle T_2_. MRI measurements suggestive of elevated inflammation and fibrosis corroborate with increased Mac-1 positive cells as well as increased Picrosirius red staining/COL1a gene expression that is returned to WT levels in response to Losartan.

**Conclusions:**

MRI is sensitive to and tightly corresponds with pathological changes in DyW mice and thus is a viable and effective non-invasive tool for assessing pathological changes.

## Introduction

Congenital muscular dystrophy (CMD) is a heterogeneous group of neuromuscular disorders that result from defects in proteins related to the dystrophin-glycoprotein complex (DGC). The DGC is an integral part of the muscle membrane and is responsible for membrane stability as well as signal transduction [1]. Laminin-deficient CMD type 1A (MDC1A) is an autosomal recessive disease caused by mutations in the LAMA2 gene that encodes for the alpha two chain of the muscle- and Scwhann cell-specific heterotrimeric extracellular matrix protein Laminin-211 [2]. Absence of a functional copy of this protein results in defective myofiber anchoring and vast signaling dysregulation that manifests in a multitude of secondary pathologies, including failed regeneration, inflammation, fibrosis, apoptosis, and necrosis [3, 4]. Children with this disease present at/soon after birth with severe weakness, atrophy and hypotonia, and ultimately die prematurely due to respiratory complications or failure to thrive [5].

The current gold standard for visualization of muscle pathology is biopsy. While informative, it provides only a limited sampling of the entire muscle and is considered invasive and potentially painful. Furthermore, effective preclinical studies often rely on histological analyses of a muscle post-euthanasia, hence establishing a non-invasive method to track therapeutic progress would be incredibly beneficial to preclinical research in addition to patient care.

Over the last two decades, magnetic resonance imaging (MRI) has emerged as an important tool in muscle research. This has been particularly true in the realm of muscular dystrophy, where it has been shown in Duchenne muscular dystrophy (DMD) that MRI is a valuable tool for in vivo monitoring of muscle health and disease progression [6, 7]. By exploiting the intrinsic MR relaxation properties, muscle size and composition can be reliably determined. For example, 3D T_1_-weighted (T_1_w) images can be used to generate high-resolution measurements of muscle volumes and cross-sectional areas while T_2_-weighted (T_2_w) MRIs can be used to further delineate between muscle, fat, and inflammation/edema [7].

The Lama2^Dyw-/+^ (DyW) mouse model is the most commonly studied animal model for MDC1A. Unlike the *mdx* mouse model frequently utilized in DMD research, DyW mice exhibit a more severe phenotype and rarely survive past 2 months [8–10]. Because of their stunted growth and frailty, these mice can present inherent challenges for MR imaging. While initial observations were made on the larger and less severe Dy/Dy model of MDC1A by Tardif de Gery *et*. *al* in 2000, the present study shows for the first time that it is not only possible to detect the underlying DyW pathology, but also that MRI can be used to detect therapeutic improvements in DyW muscles (in this case in response to the Angiotensin II type 1 receptor blocker, Losartan). Most importantly, MR indices coincide with biochemical and histological analyses and thus further validate the use of MRI as a viable and effective biomeasure for preclinical studies, even in very aggressive scenarios.

## Materials and Methods

### Animals

Heterozygous B6.129 Lama2^dy-W/+^ (DyW) mice carrying a mutation in the LAMA2 gene were kindly provided by Dr. Eva Engvall (Burnham Institute, La Jolla, CA, USA) and housed in the Laboratory Animal Care Facility at the Charles River Campus of Boston University on a 12:12 hour light-dark cycle. All animal procedures were approved by IACUC at Boston University (permit number 13–055) and conducted to minimize animal suffering at all times. Losartan was provided in the drinking water *ad libitum* (600mg/L, Cozaar by Merck pharmaceuticals with 25 g/L of sucrose to increase palatability) beginning at week two until collection at week seven [11]. Typically, animal water consumption is in the range of 1.5ml/10g/day [12]. At seven weeks of age, following 5 weeks of treatment, animals were shipped to University of Florida for skeletal muscle imaging in the Advanced Magnetic Resonance and Spectroscopy (AMRIS) facility. All animals were imaged and euthanized with overdose of Isoflurane within 24 hours of arrival. Hind limb muscles were extracted for histological and biochemical analyses and shipped back to Boston University for further analysis.

### MR acquisition

Magnetic resonance imaging (MRI) and spectroscopy (MRS) were performed in a 4.7T horizontal bore magnet (Agilent). The animals were anesthetized using an oxygen and isoflurane mixture (3% isoflurane) and maintained under 0.5–1% isoflurane for the duration of the MR procedure. Respiratory rate and body temperature of the mice were monitored for the entire duration of the scan. The lower hindlimbs of the mice were inserted up to the knee into a 2.0 cm internal diameter, custom-built solenoid ^1^H coil (200 MHz). T_2_-weighted MR multiple slice, single-spin echo images were acquired with the following parameters: repetition time (TR): 2,000ms; echo time (TE): 14ms and 40ms; FOV: 10-20mm; slice thickness: 1mm; acquisition matrix: 128 x 256; and two signal averages [13]. Hahn spin echoes were implemented to avoid the contribution of stimulated echoes in the T_2_ measurement. T_2_ was determined assuming a single exponential decay. Based on our previous work, we find that calculating T_2_ from two echoes is sufficient to differentiate between healthy and damaged muscle [13, 14]. Signal to noise ratios (SNR) were 33:1 at TE = 14ms and 12:1 at TE = 40ms. Additionally, ^1^H spectroscopic relaxometry was determined from a single voxel within the posterior muscle compartment using Stimulated Echo Acquisition Mode (STEAM) with the following parameters: typical voxel size of 1.5x3.0x1.5; repetition time (TR): 9,000ms; echo time (TE): 5, 6, 7, 8, 9, 10, 15, 20, 25, 30, 35, 40, 45, 50, 55, 60, 65, 70, 80, 90, 100, 110, 120, 130, 140, 150, 160, 170, and 200ms; mixing time (T_M_): 20ms; and number of phase cycled averages = 4. Furthermore, three-dimensional gradient echo (3D-GRE, T_1_ weighted) images were acquired at 4.7T with the following parameters: field of view: 15x15x15 mm^3^; matrix size: 256x192x96; TR/TE = 50/7ms; number of averages: 2; flip angle: 40°.

### MR analysis

Images were converted to Digital Imaging and Communication in Medicine (DICOM) format using a custom-made IDL code for Varian data (IDL, ITT Visual Information Systems, Boulder, CO). Anterior and posterior compartments were outlined on axial images of the whole limb to determine the volume of individual compartments. Furthermore, muscle T_2_ values of anterior and posterior compartment were computed and analyzed using T_2_ maps, created from two echo times (TEs 14ms and 40ms) using OsiriX (Version 3.9.4, Geneva, Switzerland), an open-source software. Muscle T_2_ was calculated from 6–8 middle MR images. Imaging-based T_2_ was calculated using the following equation: T_2_ = (26ms)/ ln (SI_14_/SI_40_), where SI_14_ and SI_40_ are the image pixel intensities at TE of 14ms and 40ms, respectively. Additionally, imaging T_2_ values of the muscle were calculated from T_2_ maps by excluding the pixels that had T_2_ values greater than 2 standard deviations above the mean muscle T_2_ value found in control mice (>27ms) (defined as hyperintense throughout the manuscript). Finally, the muscle water-only (^1^H_2_O) T_2_ data was analyzed using a custom-written software (IDL; Exelis VIS, Herndon, VA). Specifically, ^1^H_2_O relaxation time was derived from the decay in H_2_O signal at non-linear spaced echo times (TEs: 5, 6, 7, 8, 9, 10, 15, 20, 25, 30, 35, 40, 45, 50, 55, 60, 65, 70, 80, 90, 100, 110, 120, 130, 140, 150, 160, 170, and 200ms) using complex principal component analysis [7, 15]. ^1^H_2_O T_2_ was determined by a non-linear curve fitting the decay in water signal as a function of TE using a mono-exponential model [16, 17] as well as using non-negative least squares (T_2_-NNLS) [18].

### Hematoxylin and Eosin (H&E) Staining

Tibialis anterior and Gastrocnemius-Soleus muscle were embedded in TissueTek OCT compound (Sakura Finetek, Torrance, CA, USA) and frozen using isopentane chilled in liquid nitrogen. 7μM frozen sections of TA and GS were obtained from the midbelly region of the TA muscle using a Leica CM 1850 cryostat. Sections were air-dried at room temperature for 5 minutes and fixed in chilled acetone for 5 minutes. They were then hydrated through decreasing grades of alcohol and stained with hematoxylin (Fisher Scientific, Fair Lawn, NJ, USA) for 1 minute, followed by development in 1% ammonium hydroxide for 1 minute. Sections were subsequently stained with Ruben’s Eosin-Phloxine working Solution (Biocare Medical LLC) for 2 minutes. After dehydration through increasing grades of alcohol and xylene, sections were mounted using Cytoseal 280 (Richard Allen Scientific, Kalamazoo, MI, USA). Slides were imaged with a Nikon DSFi1 camera head attached to a Nikon ECLIPSE 50i light microscope system and analyzed using NIS-Elements Basic Research 3.0 software.

### Picrosirius Staining

Picrosirius Red (American MasterTech Scientific, Inc., Lodi, CA, USA) staining was performed on TA and GS according to the manufacturer’s instructions. Sections were fixed with chilled acetone for 5 minutes and then rehydrated through decreasing grades of alcohol. Rehydrated sections were stained with Picrosirius red solution for 15 minutes, rinsed twice in 0.5% acetic acid, and then dehydrated in increasing grades of alcohol and subsequently cleared in xylene. The sections were mounted using Cytoseal 280.

### Immunohistochemistry

Frozen tissue sections were fixed in acetone for 15 minutes then left to air dry for 15 minutes. Protocol for Mouse on Mouse (M.O.M.) serial immunostaining for frozen sections provided by Vector Labs (Burlingame, CA) was followed using anti-CD11b (Mac-1) (BD Biosciences, Franklin Lakes, NJ). Sections were then stained with DAPI and mounted with 2:1 Glycerol:PBS mixture and imaged with a Nikon DSFi1 camera head attached to a Nikon ECLIPSE 50i light microscope system. These images were analyzed using NIS-Elements Basic Research 3.0 software. IHC quantification was completed by averaging Mac-1 positive cells on three separate 40x field views per sample.

### Gene Expression

RNA from 25mg liquid nitrogen of snap-frozen pooled hind limb muscles (TA, GS) from individual animals was extracted with TRIzol reagent (Invitrogen, Carlsbad, CA) according to the manufacturer’s instructions. Reverse transcription was completed with the High Capacity cDNA Reverse Transcription Kit (Applied Biosystems, Foster City, CA, USA) using 1μg of RNA. Gene expression analysis was completed using TaqMan assays (Applied Biosystems, Foster City, CA, USA) on an ABI 7300 Real Time PCR system. 18s ribosomal subunit RNA served as the endogenous control and gene expression was calculated using the ΔΔCt method.

### Statistical Analysis

Statistical analyses were performed using GraphPad Prism 6 Software (GraphPad Software, La Jolla, CA, USA) and included one way analysis of variance (ANOVA) followed by Tukey’s multiple comparisons test. All data are presented as mean ± standard deviation. Statistical significance was set at p<0.05. Number of mice used in the study are as follows: WT n = 6, DyW n = 8, and DyW Losartan-treated n = 4)

## Results

### MRI is able to detect differences (or lack thereof) in muscle volume of treated and untreated DyW mice

**Fig 1:**
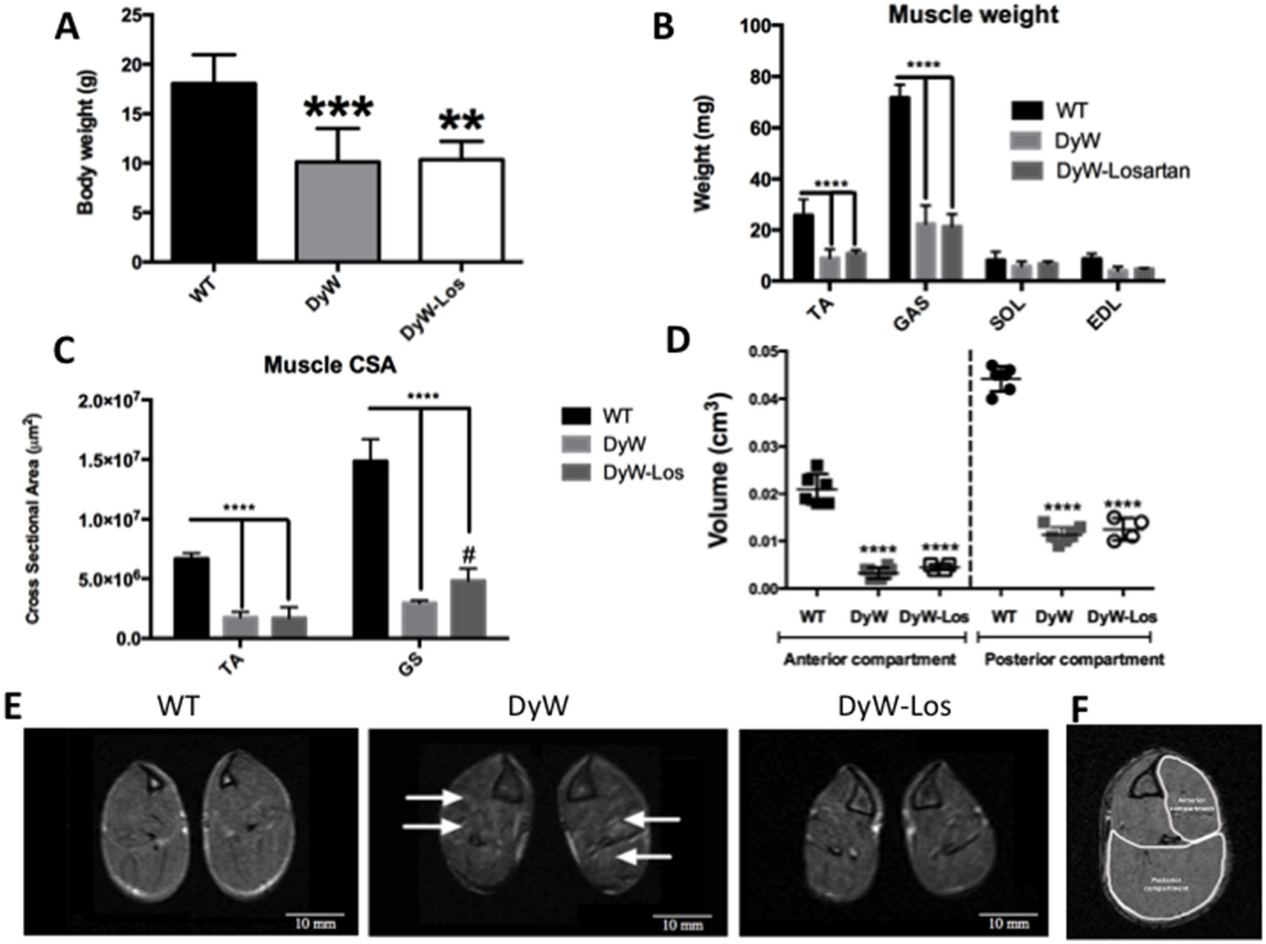
MRI is able to detect differences (or lack thereof) in DyW muscle volume. A) At 7 weeks of age, DyW mice weigh significantly less than age-matched WT littermates (p<0.001, one-way ANOVA). Losartan treatment had no effect on DyW total bodyweight. B-C) Hind limb muscles (Tibialis Anterior, Gastrocnemius, Soleus, Extensor Digotorum Longus) from untreated DyW are significantly smaller than WT muscles in terms of weight (p<0.0001, one-way ANOVA) and cross-sectional area (CSA) (p<0.0001, one-way ANOVA). Losartan treatment had no effect on TA muscle weight/CSA but did result in a significant increase in GS CSA (p<0.05, one-way ANOVA). D) 3D T_1_-weighted MR analysis shows that both anterior and posterior compartments of DyW hind limb muscle are significantly smaller in terms of volume than WT mice (p<0.0001, one-way ANOVA). Similar to total bodyweight, Losartan had no effect on either anterior or posterior compartment muscle volume. E) MR images of WT, DyW, and Losartan-treated DyW mice show that DyW muscle is much smaller than WT and exhibit multiple areas of hyperintensity (indicated by arrows) on T2 weighted MR images. F) Outline of anterior and posterior compartments. (* is used to denote significance between WT and DyW; # is used to denote significance between DyW and DyW Losartan-treated; * = p<0.05, ** = p<0.01, *** = p<0.001, **** = p<0.0001, this also applies to the other symbols). WT n = 6, DyW n = 8, DyW Losartan-treated n = 4.

It has been previously shown that Losartan does not have a significant effect on bodyweight in DyW mice [19]. Indeed, at 7 weeks of age, we did not observe any difference in body weight between untreated DyW (10.11±3.39g) and Losartan-treated DyW mice (10.35 ±1.85g) with both groups being significantly smaller than age-matched WT littermates (18.05 ± 2.91g) (p<0.05, one-way ANOVA) (Fig 1A). The same trend was seen for muscle size; MR images qualitatively show that DyW mice have much smaller hind limb muscles as well as many areas of hyperintense pixels on T_2_-weighted images not evident in WT muscle. While Losartan-treated hind limbs do not exhibit increases in overall muscle size, they do show attenuation of MR hyperintense areas compared to untreated DyW mice (Fig 1E). To quantify muscle size, 3D T_1_ weighted MRI was utilized. Muscle volumes of both anterior and posterior hind limb compartments of DyW mice were significantly smaller than WT counterparts (p<0.0001, one-way ANOVA) (Fig 1D). Losartan did not induce increased muscle volume in either compartment compared to untreated DyW mice, which is also reflected in muscle weights and cross-sectional areas (Fig 1B and 1C). These results firstly confirm, using MRI, what previous studies have shown that Losartan does not induce change in overall size/muscle weight [20]. Secondly, 3D MRI can readily detect differences (or lack thereof) in muscle size in mice as small as DyW.

### MR quantifications are sensitive to inflammatory and fibrotic attenuation in DyW mice and coincide with histological/biochemical analyses

**Fig 2:**
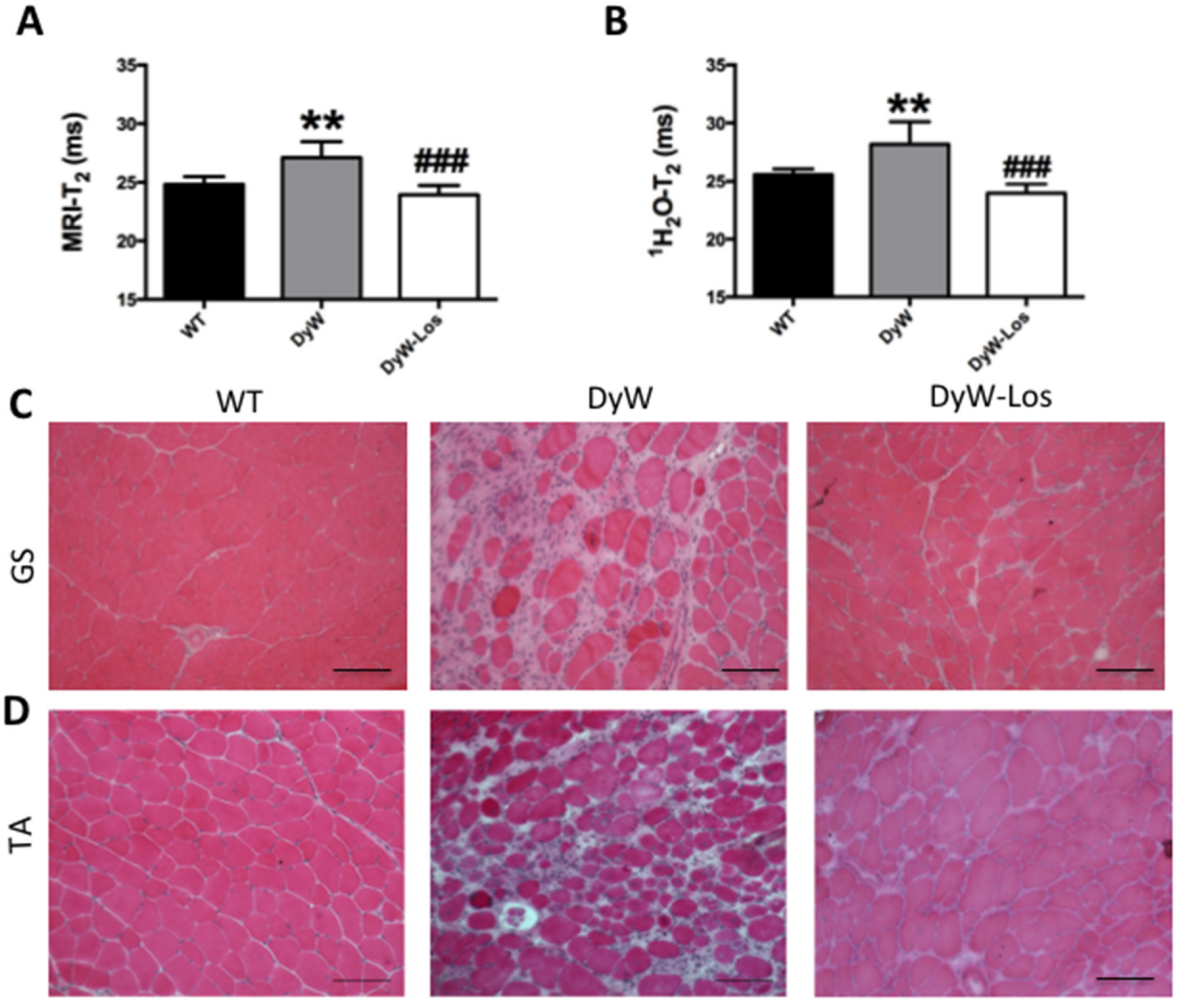
MR analyses are sensitive to and coincide with inflammatory pathology and resolution in DyW mice. A) T_2_ weighted MRI of the entire DyW posterior compartment hind limb muscles is significantly elevated compared to age-matched WT littermates (p<0.01, one-way ANOVA) indicative of inflammation/edema. Losartan treatment significantly reduced T_2_ values to WT levels (p<0.001, one-way ANOVA). B) DyW mice also exhibit significantly greater localized ^1^H_2_O T2 levels compared to WT counterparts (p<0.01, one-way ANOVA) which was also significantly lowered in response to Losartan treatment (p<0.001, one-way ANOVA). C-D) Histological analyses verified elevated T_2_ levels were due to muscle inflammation and edema. H&E staining shows extensive infiltration of inflammatory cells in DyW muscle compared to WT in both GS (C) and TA (D). Note: H&E images taken at 20x magnification. (* is used to denote significance between WT and DyW; # is used to denote significance between DyW and Losartan-treated DyW; * = p<0.05, ** = p<0.01, *** = p<0.001, **** = p<0.0001, this also applies to the other symbols). WT n = 6, DyW n = 8, DyW Losartan-treated n = 4.

It has been shown that DyW mice have a severe inflammatory pathology due to large numbers of infiltrating cells [21–23]. As previously stated, it can be seen in MR images that DyW mice exhibit many areas of hyperintensity (indicative of areas of inflammation and edema) [24] that are not evident in WT muscle, and that Losartan-treated hind limbs do not show the same extent of T_2_ hyperintensity (Fig 1E). Interestingly, Losartan treatment has been shown to reduce inflammatory cell infiltration [11, 19]. Quantification of DyW posterior compartment muscle T_2_ showed a significantly elevated T_2_ compared to WT (Fig 2A) (27.10 ± 1.38ms vs. 24.83 ± 0.66ms; p<0.01, one-way ANOVA). Following treatment with Losartan, we observed a significant decrease in muscle T_2_ values between DyW and DyW-Losartan-treated mice (27.10 ± 1.38ms vs. 23.92 ± 0.80ms; p<0.001, one-way ANOVA). This was further confirmed using highly TE sampled, localized ^1^H_2_O MRS quantification, which demonstrated a similar trend thereby validating the use of MR as a non-invasive tool to detect differences in inflammatory pathology in DyW mice (Fig 2B) using both imaging and spectroscopic methods.

**Fig 3:**
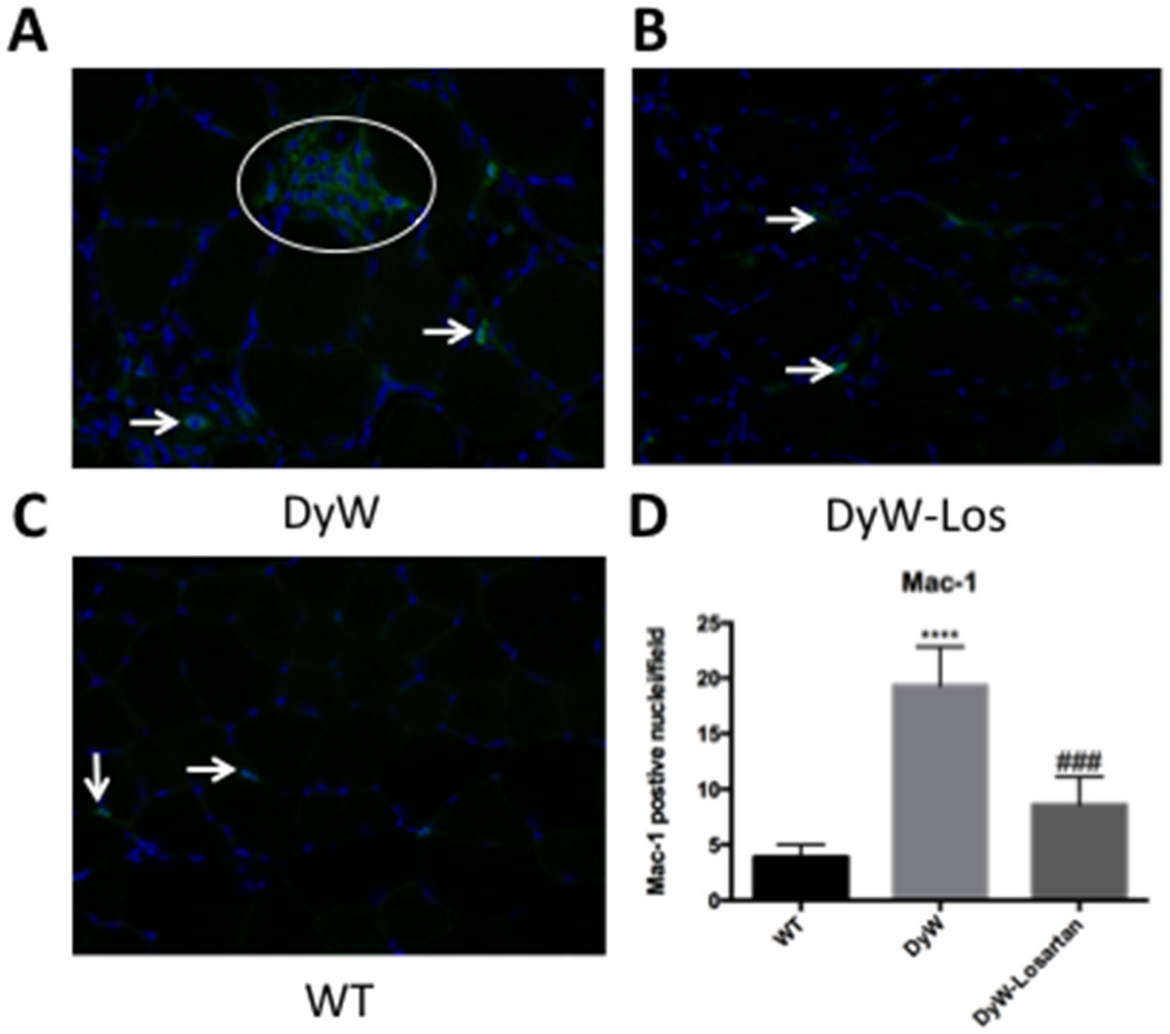
Mac-1 staining confirms inflammatory resolution suggested by MRI quantifications in Losartan-treated DyW mice. A-D) Mac-1 (anti-CD11b) staining shows that there is a significant increase in Mac-1 positive cells in DyW muscle compared to WT (p<0.0001, one-way ANOVA). Losartan treatment significantly reduced the amount of Mac-1 positive cell infiltration in DyW muscle (p<0.001, one-way ANOVA). (* is used to denote significance between WT and DyW; # is used to denote significance between DyW and Losartan-treated DyW; * = p<0.05, ** = p<0.01, *** = p<0.001, **** = p<0.0001, this also applies to the other symbols). WT n = 6, DyW n = 8, DyW Losartan-treated n = 4.

Changes in T_2_ values were compared with histological and biochemical analyses of the same tissues. H&E staining shows DyW mice have large areas of inflammation and edema with infiltrating cells in both posterior compartment (GS) (2C), and anterior compartment muscle (TA) (Fig 2D) whereas these areas are absent in WT mice. Muscles of DyW mice also have significantly more Mac-1-positive cells (Fig 3A–3D) (3.915 vs. 19.33, p<0.0001, one-way ANOVA). In response to Losartan treatment, the number of Mac-1-positive infiltrating cells are significantly reduced back to WT levels (19.33 (DyW) vs. 8.585 (DyW-Los) vs. 3.915 (WT) p<0.001, one-way ANOVA). These analyses corroborate the fact that elevated T_2_ values in DyW muscle may in fact be due to increased inflammation, which are attenuated in response to Losartan.

**Fig 4:**
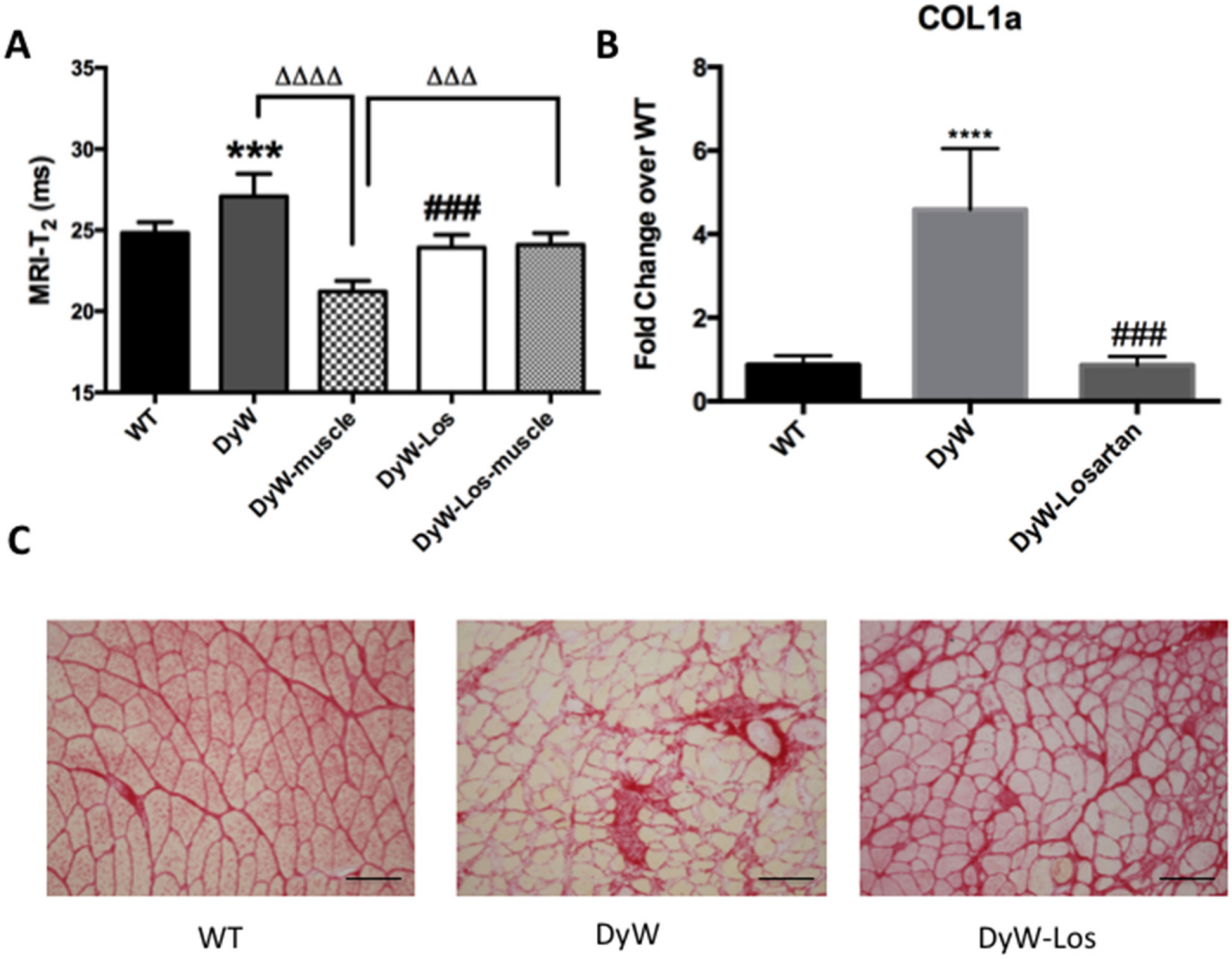
MR analyses are sensitive to and correlate with fibrotic pathology and resolution in DyW mice. A) When areas of hyperintensity are removed from whole posterior compartment T_2_ levels, DyW mice show significantly reduced T_2_ levels compared to WT (p<0.0001, one-way ANOVA). Mice treated with Losartan do not exhibit this reduction of T_2_ values following removal of hyperintense areas. B) Because reduced T_2_ levels are indicative of fibrosis, specifically collagen buildup, qRT-PCR was used to assess COL1a gene expression. DyW mice exhibit significantly greater gene expression of COL1a compared to age-matched WT littermates (p<0.0001, one-way ANOVA), which was rescued back to WT levels in response to treatment with Losartan (p<0.001, one-way ANOVA). C) Picrosirius red staining of GS confirms extensive fibrosis in DyW mice that is rescued by Losartan treatment. Picrosirius red images taken at 20x. (* is used to denote significance between WT and DyW; # is used to denote significance between DyW and DyW Losartan-treated; Δ is used to denote significance between DyW and DyW-muscle (with removal of hyperintensive areas); & is used to denote significance between DyW-muscle and DyW Losartan-treated muscle; * = p<0.05, ** = p<0.01, *** = p<0.001, **** = p<0.0001, this also applies to the other symbols). WT n = 6, DyW n = 8, DyW Losartan-treated n = 4.

Additionally, it has been established that Losartan is also a potent anti-fibrotic agent [19, 25, 26]. A pixel-by-pixel analysis of DyW T_2_ maps indicated that muscle T_2_ values were significantly lower when only considering muscle pixels without areas of T_2_ elevation (27.10 ± 1.38ms vs. 21.20 ± 0.68ms; p<0.0001, one-way ANOVA), suggestive of fibrosis (Fig 4A) [27]. In the case of Losartan-treated mice, pixel-by-pixel analysis of the muscle pixels without T2 elevation, revealed that muscle T_2_ values were returned to WT levels (21.20 ± 0.68ms vs. 23.91 ± 0.84ms; p<0.001, one-way ANOVA) (Fig 4A) thus demonstrating that MR indices can also detect differences in fibrosis in DyW mice in addition to inflammation. The reduction of fibrosis suggested by normalized muscle-specific T_2_ values in response to Losartan was confirmed with Picrosirius red staining which shows markedly increased fibrotic tissue in DyW mice compared to WT (Fig 4C). These results were further supported by COL1a overexpression in DyW mice (p<0.0001, one-way ANOVA) that returned to WT levels in response to Losartan (p<0.001, one-way ANOVA) (Fig 4B).

## Discussion

In this study, MRI was utilized to monitor changes in muscle pathology and size in DyW mice in response to treatment with the Angiotensin II type I receptor blocker Losartan. Based on T_1_-weighted MR analyses, Losartan treatment did not result in muscle volume changes in DyW mice. On the other hand, Losartan treatment did normalize muscle T_2_ values to WT levels. These findings were further confirmed by histological and biochemical analysis showing that MR measurements are sensitive to and reflect therapeutic improvements in DyW muscles.

Losartan has been shown to exhibit both anti-inflammatory and anti-fibrotic effects. Muscle T_2_ has been shown to be elevated during inflammation [24] and decreased with fibrosis [27]. In this study we found MR evidence of both decreased inflammation and fibrosis in Losartan-treated DyW muscle. Compared to untreated DyW mice, Losartan-treated mice had decreased total muscle compartment T_2_ values suggesting a decrease in inflammation following Losartan treatment. This was confirmed by a reduction in Mac-1 positive cells in treated DyW muscles. Moreover, it has been shown that fibrotic lesions—more specifically lesions with large amounts of collagen buildup—have a lower T_2_ signal intensity [28–30]. Cardiac MRI studies in DMD subjects have reported an age related decrease in myocardial T_2_ compared to controls [31, 32] and an increase in myocardial T_2_ heterogeneity [33]. Similar results have been observed in animal models with diabetic induced cardiac fibrosis [27, 34]. The trend of decreased T_2_ levels due to fibrosis was also seen in the current MRI examination of DyW hind limb skeletal muscle. When pixels with elevated T_2_ values were excluded from the T_2_ analyses, muscle T_2_ values in DyW mice demonstrated a significant decrease, suggestive of fibrotic tissue buildup [35]. Interestingly, when DyW mice were treated with Losartan, muscle T_2_ values were returned to WT levels. These observations were validated by both histological and biochemical analyses that demonstrated Losartan-treated mice exhibit significantly less COL1a gene expression as well as decreased interstitial fibrosis as seen with Picrosirius red staining.

The MR results presented in this study firstly corroborate previous publications demonstrating the positive effects of Losartan in dystrophic models [20, 36]. We previously found and have replicated in this study that Losartan does not have a measurable effect on total body or muscle weight but does ameliorate fibrotic and inflammatory pathologies. This suggests that an anabolic therapy should be utilized in conjunction with Losartan treatment to achieve comprehensive pathological attenuation. We have previously shown that Insulin-like Growth Factor-1 (IGF-1) overexpression can result in increased muscle mass [35]. In yet another study it has been shown that Adeno-Associated Virus-driven over-expression of IGF-1 can be monitored using MRI muscle measures [37].

The current study provides proof of concept that MRI can quantify muscle mass and composition in small animal models and is able to detect changes in muscle pathology that concur with histological and biochemical analyses, and thus should be considered as a viable and effective non-invasive method of assessing therapeutic efficacy in preclinical studies.
